# A Treatise on Therapeutics, Comprising Materia Medica and Toxicology

**Published:** 1874-08

**Authors:** 


### Books Reviewed.

*A Treatise on Therapeutics, comprising Materia Medica and Toxicology, with especial reference to the application of the Physiological Action of Drugs to Clinical Medicine.* By H. C. Wood, Jr., M. D., etc. Piladelphia: J. B. Lippincott & Co., 1874. Buffalo: Martin Taylor.

Dr. Wood's treatise is one of the fruits of a new era in Medicine, an era in which physiological facts are the standpoint from which therapeutical observations are made. Empiricism, or as our author would less harshly state it, clinical experience, has heretofore been the method by which a knowledge of therapeutics has been gained. The early practitioners of medicine told us that ergot would cause uterine contraction, but they had only learned the fact by observation, perhaps by accident, and could not tell its mode of action or anything concerning its physiological effects beyond what they saw in their clinical observations; they were, consequently, ignorant of many of its present therapeutic uses. No one has been more active in the physiological investigation of therapeutical agents than has Dr. Wood, and the work before us is the result of his studies and experiments. He tells us in his preface that he has endeavored to sift the true from the false and present to his readers the many scattered facts concerning the physiological action of medicine.

The introduction briefly explains the terms used in Pharmacy and Materia Medica, and gives the various modes of administering drugs.

The Materia Medica is divided into two great divisions: First, substances which act on the solids and fluids of the body. Second, substances which act externally to the body. The first division is sub-divided into general remedies as: astringents, tonics, antispasmodics, analgesics, etc.; and Local remedies, as emetics, cathartics, diuretics, etc; the second division includes antacids, anthelmintics, digestants, etc.

The physiological action of each drug is considered in detail, so that the reader is at once led to consider for himself its therapeutical application. Students who use Dr. Wood's treatise as a text book will find that much of the irksomeness which has hitherto been supposed to attend the study of Materia

Medica is done away with; they will find, if they are in earnest in the pursuit of knowledge, that the author has opened up to them a store house of wisdom. Dr. Wood's work deserves a place in the library of every educated physician; it will be found to be a most valuable addition to medical literature.

